# Usefulness of an accelerated transoesophageal stress echocardiography in the preoperative evaluation of high risk severely obese subjects awaiting bariatric surgery

**DOI:** 10.1186/1476-7120-8-30

**Published:** 2010-07-28

**Authors:** Sylvie Legault, Mario Sénéchal, Sébastien Bergeron, Marie Arsenault, Michel Tessier, Jean Guimond, Paul Poirier

**Affiliations:** 1 Department of cardiology, Institut Universitaire de Cardiologie et de Pneumologie de Québec, Quebec, Canada; 2 Department of medical Imaging, Institut Universitaire de Cardiologie et de Pneumologie de Québec, Canada; 3 Faculty of pharmacy, Laval University, Quebec, Canada

## Abstract

**Background:**

Severe obesity is associated with an increased risk of coronary artery disease (CAD). Bariatric surgery is an effective procedure for long term weight management as well as reduction of comorbidities. Preoperative evaluation of cardiac operative risk may often be necessary but unfortunately standard imaging techniques are often suboptimal in these subjects. The purpose of this study was to demonstrate the feasibility, safety and utility of transesophageal dobutamine stress echocardiography (TE-DSE) using an adapted accelerated dobutamine infusion protocol in severely obese subjects with comorbidities being evaluated for bariatric surgery for assessing the presence of myocardial ischemia.

**Methods:**

Subjects with severe obesity [body mass index (BMI) >40 kg/m^2^] with known or suspected CAD and being evaluated for bariatric surgery were recruited.

**Results:**

Twenty subjects (9M/11F), aged 50 ± 8 years (mean ± SD), weighing 141 ± 21 kg and with a BMI of 50 ± 5 kg/m^2 ^were enrolled in the study and underwent a TE-DSE. The accelerated dobutamine infusion protocol used was well tolerated. Eighteen (90%) subjects reached their target heart rate with a mean intubation time of 13 ± 4 minutes. Mean dobutamine dose was 31.5 ± 9.9 ug/kg/min while mean atropine dose was 0.5 ± 0.3 mg. TE-DSE was well tolerated by all subjects without complications including no significant arrhythmia, hypotension or reduction in blood arterial saturation. Two subjects had abnormal TE-DSE suggestive of myocardial ischemia. All patients underwent bariatric surgery with no documented cardiovascular complications.

**Conclusions:**

TE-DSE using an accelerated infusion protocol is a safe and well tolerated imaging technique for the evaluation of suspected myocardial ischemia and cardiac operative risk in severely obese patients awaiting bariatric surgery. Moreover, the absence of myocardial ischemia on TE-DSE correlates well with a low operative risk of cardiac event.

## Background

Severe obesity is associated with multiple comorbidities and an increased risk for coronary artery disease (CAD) since many risk factors for CAD are present in obese patients. Bariatric surgery is probably the most effective procedure for long term weight management as well as reduction of comorbidities in this population [1]. It also increases survival [2]. Preoperative evaluation of suspected myocardial ischemia and cardiac operative risk may be necessary in these high risk patients but unfortunately standard imaging techniques are often suboptimal in severely obese subjects [3]. Hansen et al. have reported that the accuracy of thallium-201 single-photon emission computed tomography is reduced in obesity [4]. On the other hand, transthoracic dobutamine stress echocardiography has a high sensitivity and specificity for the detection of myocardial ischemia [5]. This imaging technique is also used for the stratification of preoperative risk of cardiac events [6,7]. Unfortunately standard transthoracic stress echocardiography is often suboptimal in obese patients because of paucity of echogenicity.

Transesophageal echocardiography provides superior quality cardiac imaging and transesophageal dobutamine stress echocardiography (TE-DSE) compares favourably with myocardial scintigraphy for the detection of myocardial ischemia in non severely obese patients [8]. A study of 341 obese patients body mass index (BMI) > 27,5 kg/m^2 ^demonstrated that transesophageal echocardiography is a safe and well tolerated procedure in this population [9]. Moreover, in a study evaluating 23 severely obese patients, Madu showed that TE-DSE is a useful and safe method for the detection of CAD [10] while a small study in 7 severely obese patients revealed that TE-DSE is a valid non-invasive imaging technique for evaluating cardiac risk before bariatric surgery [11]. The purpose of this study was to demonstrate the safety and reliability of a new adapted transoesophageal accelerated dobutamine infusion protocol including atropine for assessing cardiac ischemia.

## Methods

Twenty severely obese patients (BMI > 40 kg/m^2^) were recruited between March 2006 to June 2007 from the bariatric surgery preoperative evaluation clinic. All patients were considered at high cardiovascular risk because of known CAD or suspected CAD on the basis of an abnormal resting ECG or the presence of at least 2 risk factors for CAD (systemic hypertension, diabetes mellitus, smoking, dyslipidemia, positive family history of premature CAD or sleep apnea). Exclusion criteria included: recent acute coronary syndrome, uncompensated heart failure, uncontrolled ventricular or supraventricular arrhythmias, oral or oesophageal pathology, uncontrolled systemic hypertension or significant valvular heart disease. Twenty patients underwent a TE-DSE. Beta-blockers were stopped 48 hours before the stress test. The study was approved by the local hospital ethics committee in accordance with the Helsinki declaration and subjects gave signed informed consent.

### TE-DSE protocol

TE-DSE was performed with a commercially available echocardiography machine (Sonos 5500, Hewlett-Packard) using a multiplane probe. Antianginal medications were discontinued for at least 24 hours prior the study. Studies were performed after an overnight fast with patients in the left lateral decubitus position. Topical oropharyngeal anesthetic spray was used and light sedation was achieved with the use of intravenous midazolam (Midazolam Injection, Sandoz, Canada) or diazepam (Diazemul, Pfizer, Canada) before the introduction of the transesophageal probe. All patients received supplemental oxygen through nasal prongs. Oxygen saturation was continuously monitored throughout the procedure using pulse oxymetry. A continuous 12-lead electrocardiogram monitored for arrhythmias or ST-segment modifications suggestive of myocardial ischemia. Blood pressure was measured non-invasively every 3 minutes throughout the procedure.

Dobutamine was intravenously infused using an accelerated protocol with a starting dose of 10 ug/kg/min and increased every 2 minutes to 20, 30 and 40 ug/kg/min. Atropine sulphate was given intravenously in increments of 0.2 mg from the second stage of the dobutamine infusion (20 ug/kg/min) until 85% of the maximal heart rate (i.e. 220-age) was reached or up to a maximum of 1 mg. Images were obtained at each stage in the following sequence: 1) midesophageal four-chamber view at 0°, 2) midesophageal two-chamber view at 60°, 3) midesophageal three-chamber view at 120° and, 4) transgastric midpapillary short-axis view at 0° (see additional file 1, 2, 3, 4, 5, 6, 7, &8). Left ventricular wall motion was qualitatively assessed and myocardial ischemia was documented if a new wall motion abnormality appeared. Echocardiographies were reviewed by two echographists who were blinded to the results of the TE-DSE.

Patient's charts were reviewed for the presence of CAD risk factors, current medications and recent blood chemistry. The outcome following bariatric surgery, including the presence of perioperative cardiovascular complications, was noted. Cardiovascular complications were assessed for 30 minutes post imaging procedures. Troponin levels were measured 24-hrs post bariatric surgery.

### Statistical analysis

Population characteristics and echocardiographic data are reported as mean ± standard deviation or a proportion of the sample size.

## Results

The accelerated dobutamine infusion protocol used was well tolerated. Eighteen (90%) subjects reached their target heart rate with a mean intubation time of 13 ± 4 minutes. Mean dobutamine dose was 31.5 ± 9.9 ug/kg/min while mean atropine dose was 0.5 ± 0.3 mg. Twenty patients (55% female) with a mean age of 50 ± 8 years and mean BMI of 50 ± 5 kg/m^2 ^were recruited. Only 1 patient was known to have CAD (previous coronary bypass surgery) but the majority of subjects (74%) had a least 3 risk factors for CAD and an abnormal metabolic profile (Table 1). Six patients (30%) had abnormal resting ECGs showing abnormal Q waves (2 patients), abnormal rightward axis (1 patient) or non-specific conduction abnormalities (3 patients).

**Table 1:** Study population characteristics (n = 20)

Age (years)	50 ± 8
**Men/Women**	9 (45%)/11 (55%)

**Weight (kg)**	141 ± 21

**BMI (kg/m2)**	50 ± 5

**Known CAD**	1 (5%)

**Risk factors for CAD**	
Systemic hypertension	15 (75%)
Diabetes mellitus	13 (65%)
Smoking (active or past)	8 (40%)
Dyslipidemia	13 (65%)
Positive family history for CAD	3 (15%)
Sleep apnea	15 (75%)

**Abnormal resting ECG**	6 (30%)

**Medications**	
Aspirin	10 (50%)
Beta-blockers	5 (25%)
Calcium channel blockers	5 (25%)
ACEI/ARB	12 (60%)
Statins	6 (30%)
Fibrates	2 (10%)
Ezetimide	2 (10%)
Nitrates	1 (5%)
Diuretics	8 (40%)
Oral hypoglycaemic agents	9 (45%)
Insulin	5 (25%)

**Baseline blood chemistry**	
Fasting blood glucose (mmol/L)	7.4 ± 3,5
HbA1c (%)	6.9 ± 1.5
Total cholesterol (mmol/L)	4.7 ± 1.4
LDL-cholesterol (mmol/L)	3.1 ± 1.2
HDL-cholesterol (mmol/L)	1.3 ± 0.2
Total cholesterol/HDL cholesterol	3.7 ± 1.3
Triglycerides (mmol/L)	2.0 ± 1.3

ACEI = angiotensin-converting enzyme inhibitors, ARB = angiotensin receptor blockers, BMI = boby mass index, CAD = coronary artery disease, HbA1c = glycated haemoglobin (normal range 4-6%), HDL = high-density lipoprotein, LDL = low-density lipoprotein

TE-DSE was well tolerated by all subjects with no complications including no significant arrhythmia, hypotension or reduction in blood arterial saturation. No patient develops clinical pneumonia-induced aspiration. Sedatives were administered to ensure subject's comfort (midazolam 10 ± 7 mg or diazepam 15 ± 6 mg). Seventeen patients (85%) required atropine to reach their target heart rate. Only 7 subjects (35%) had adequate transgastric images for the evaluation of left ventricular wall motion but transesophageal images were of diagnostic quality in all patients. At peak dose, 3 subjects complained of symptoms suggestive of myocardial ischemia. One patient developed ECG changes suggestive of myocardial ischemia (ST segment depression ≥ 1 mm) and 2 patients had new left ventricular wall motion abnormalities compatible with myocardial ischemia (hypokinesia) (Table 2). Ischemic threshold was documented in both patients.

**Table 2:** Transesophageal dobutamine echocardiography results (n = 20)

Resting HR (bpm)	78 ± 12
Peak dose HR (bpm)	147 ± 12

Resting SBP (mmHg)	140 ± 19

Peak dose SBP (mmHg)	168 ± 36

Resting DBP (mmHg)	83 ± 9

Peak dose DPB (mmHg)	89 ± 15

Symptoms of myocardial ischemia	3 (15%)

ECG changes suggestive of ischemia	1 (5%)

New LV wall motion abnormalities	2 (10%)

DBP = diastolic blood pressure, HR = heart rate, LV = left ventricular, SBP = systolic blood pressure

## Discussion

The main finding of this study is that TE-DSE using an accelerated dobutamine protocol is a safe and well tolerated imaging technique for the evaluation of suspected myocardial ischemia in severely obese patients awaiting bariatric surgery.

In this study, we used an accelerated protocol for the infusion of dobutamine. The purpose was to minimize probe insertion time. Our probe insertion time of 13 ± 4 min compares favourably with previous studies. In the study by Madu et al [10], probe insertion time was 29 ± 7 min and significant hypotensive response was noted in 43% of their subjects. Our accelerated protocol was very well tolerated with no significant hypotension or reduction in oxygen saturation and 90% of our patients reached their target heart rate. Pastorius et al. demonstrated the tolerability of an accelerated dobutamine infusion in standard transthoracic dobutamine echocardiography [12]. To the best of our knowledge, the present study is the first to have used an accelerated protocol in TE-DSE.

Obesity has become an epidemic condition around the world and is associated with increased risk of CAD and risk factors for CAD (i.e. systemic hypertension, diabetes, dyslipidemia and sleep apnea) [13,14]. In the United States, the percentage of adults who are obese (BMI > 30 kg/m^2^) was estimated at 23.9% in 2005 with approximately 4.8% considered to be severely obese (BMI > 40 kg/m^2^) [14]. The BMI subgroups experiencing the most rapid growth are ≥ 35 or ≥ 40 kg/m^2 ^[13]. Bariatric surgery induces long term significant weight loss with a meta-analysis showing that the majority of patients with diabetes, dyslipidemia, systemic hypertension or obstructive sleep apnea experience complete resolution or improvement [13]. Moreover, recent studies have showed reduced cardiovascular mortality following bariatric surgery [2,15]. Preoperative evaluation prior to surgery is important in high risk patients. Of note, specific approaches regarding pre-operative risk stratification in severely obese patients have not been addressed in previous European or American Guidelines. The only tentative algorithm has been published earlier [3]. This is of importance since older and sicker patients are now being considered for bariatric surgery as in our study. Specific contraindications to bariatric surgery are few [3]. A recent study of 52 patients with known clinical CAD undergoing bariatric surgery reported a trend for more nonfatal cardiac events in comparison to patients without CAD [16]. Also, ejection fraction may be reduced in severely obese patients secondary to hibernating myocardium due to significant CAD or the presence of diabetic or obesity cardiomyopathy [3,17]. Thus, careful selective cardiac investigation to optimize medical management prior surgery and to identify potential perioperative or postoperative complications may be necessary [3]. However, the most appropriate method to assess the presence of CAD in this population remains uncertain. The role of alternative techniques in the assessment of pre operative risk such as adenosine magnetic resonance imaging or coronary computed tomography is severely obese patients is not known. We undertook the present study to demonstrate the feasibility and safety of an adapted TE-DSE in severely obese subjects.

Obese patients have a higher prevalence of systemic hypertension, CAD, and sleep apnea which could potentially increase the risk of complications during transoesophageal echocardiography. Also higher doses of sedatives are often necessary in this population which could further increase the risk of complications, including oxygen desaturation. In a study comparing transesophageal echocardiography in 341 obese subjects (mean BMI 41 kg/m^2^) with 323 nonobese subjects, Garimella et al showed that the frequency of complications did not differ between both groups. Oxygen desaturation was infrequent when supplemental oxygen was used [9]. Another study demonstrated that the frequency of complications seen during TE-DSE in obese subjects (mean BMI 39 kg/m^2^) with suspected CAD were not significantly different from those of nonobese individuals [18]. In our study, despite a higher BMI (mean BMI 50 kg/m^2^) and the use of large doses of sedatives, there were no complications including no significant hypotension or oxygen desaturation reinforcing the safety of TE-DSE in severely obese subjects. Only 35% of our subjects had adequate transgastric imaging. We propose that severe abdominal obesity might displace the stomach in a more anterior position resulting in altered probe contact. In fact, in a previous study of TE-DSE in nonobese subjects, 8% of patients had poor transgastric echocardiographic images related to the presence of hiatal hernia or gastroesophageal disease [19].

Assuming equal technical competence, the debate persists regarding which imaging modality between stress echocardiography and stress myocardial perfusion imaging is the most accurate for the diagnosis of CAD. In nonobese populations, TE-DSE has showed to be a useful technique in the evaluation of CAD with a sensitivity of 82 to 92% and a specificity of 93% when compared with coronary angiography [19,20]. In patients with poor imaging windows, it has been demonstrated that TE-DSE offers better sensitivity, specificity and positive predictive value compared to transthoracic imaging [21]. In a study evaluating 23 severely obese patients with chest pain with TE-DSE, Madu showed that 7 of 9 patients with positive TE-DSE had objective confirmatory evidence of CAD either by cardiac catheterization or clinical event [10]. There are fewer reports regarding the accuracy of myocardial perfusion imaging in severely obese patients. One study [4] using Thallium-201 myocardial perfusion imaging demonstrated lower accuracy in patients with BMI > 30 kg/m^2^, and comparing single photon emission computed tomography (SPECT) results with positron emission tomography (PET), discordance was encountered with greater frequency in obese patients, mostly in the territories supplied by left anterior descending or right coronary arteries, as a result of more prominent attenuation artefacts [22]. In a study by Bhat et al., 7 severely obese patients were evaluated with TE-DSE prior to bariatric surgery to determine cardiac risk. Six of 7 patients had no evidence of ischemia. All patients successfully underwent bariatric surgery without cardiac events [11]. This compares to our study in which all patients, including both patients with abnormal TE-DSE, have undergone surgery without cardiovascular complications. Most patients in our study would be assigned to a low preoperative risk group, which is somewhat in agreement with the high normalcy rate of TE-DSE. Only one patient in our study underwent coronary angiogram. This patient was known to have atypical chest pain and TE-DSE was negative. Angiography was within normal limits in this patient.

There are some limitations inherent to our study. Without information on coronary arterial wall and lumen, for example by means of intravascular ultrasound or quantitative coronary angiography, it is speculative to comment on the accuracy of TE-DSE regarding the presence of clinically significant CAD in these patients (namely 50% stenosis on coronary angiography). At the very least, a larger study including more patients with coronary angiography should be performed before we can conclude on the validity of TE-DSE for the diagnosis of CAD in patients with severe obesity. Also since this study included only 20 patients, a larger study should be performed to assess and confirm the safety of this accelerated protocol of TE-DSE before its wide implantation in clinical practice.

## Conclusions

In conclusion, TE-DSE with an accelerated protocol is a safe and well tolerated imaging technique for the evaluation of suspected myocardial ischemia and cardiac operative risk in severely obese patients awaiting bariatric surgery. Moreover, the absence of myocardial ischemia on TE-DSE correlates well with a low operative risk of cardiac event.

## Competing interests

The authors declare that they have no competing interests.

## Authors' contributions

PP conceived the study, participated in its design and coordination and helped to draft the manuscript, he also performed statistical analysis. SL and MS participated in the design of the study, participated in the interpretation of the results and manuscript drafting. SB, MS, and MA performed TE-DSE measurements, participated in data collection, plots and calculations from ultrasound data. MT and JG participated in data collection and in the preparation of the manuscript. All authors read and approved the final manuscript.

## Supplementary Material

Additional file 1 **Video1 Rest 2 chamber**. 2 chamber view TE-DSE (rest). Click here for file

Additional file 2 **Video2 30 mg 2 chamber**. 2 chamber view TE-DSE (30 mg). Click here for file

Additional file 3 **Video3 Rest 3 chamber**. 3 chamber view TE-DSE (rest). Click here for file

Additional file 4 **Video4 30 mg 3 chamber**. 3 chamber view TE-DSE (30 mg). Click here for file

Additional file 5 **Video5 Rest 4 chamber**. 4 chamber view TE-DSE (rest). Click here for file

Additional file 6 **Video6 30 mg 4 chamber**. 4 chamber view TE-DSE (30 mg). Click here for file

Additional file 7 **Video7 Rest short axis**. short axis view TE-DSE (rest). Click here for file

Additional file 8 **Video8 30 mg short axis**. short axis view TE-DSE (30 mg). Click here for file
